# Single Fault Diagnosis Method of Sensors in Cascade System Based on Data-Driven

**DOI:** 10.3390/s21217340

**Published:** 2021-11-04

**Authors:** Wenbo Na, Siyu Guo, Yanfeng Gao, Jianxing Yang, Junjie Huang

**Affiliations:** College of Mechanical and Electrical Engineering, China Jiliang University, Hangzhou 310018, China; hznwb@cjlu.edu.cn (W.N.); gaoyanfeng@cjlu.edu.cn (Y.G.); 1800103209@cjlu.edu.cn (J.Y.); 1800103211@cjlu.edu.cn (J.H.)

**Keywords:** cascade system, fault diagnosis, sensor, data-driven

## Abstract

The reliability and safety of the cascade system, which is widely applied, have attached attention increasingly. Fault detection and diagnosis can play a significant role in enhancing its reliability and safety. On account of the complexity of the double closed-loop system in operation, the problem of fault diagnosis is relatively complex. For the single fault of the second-order valued system sensors, a real-time fault diagnosis method based on data-driven is proposed in this study. Off-line data is employed to establish static fault detection, location, estimation, and separation models. The static models are calibrated with on-line data to obtain the real-time fault diagnosis models. The real-time calibration, working flow and anti-interference measures of the real-time diagnosis system are given. Experiments results demonstrate the validity and accuracy of the fault diagnosis method, which is suitable for the general cascade system.

## 1. Introduction

Currently, the control system is showing a trend of complexity and large-scale. Along with this phenomenon, there are various types of system failures. The occurrence of the fault will cause the behavior of the control system to deviate from the regular running track inevitably. As a result, the performance of the system will be weakened, unstable, and even severe accidents such as property loss and casualties will occur. Since the 1970s, the emergence and development of fault detection and diagnosis technology have opened up a new way to ensure the safety and reliability of the system. It has appeal to growing scholars at home and abroad [1,2,3].

In practical engineering, the equipment operation data reveals the working state of the system. It is feasible for us to diagnose the status [4] and judge whether the system faults in time via collecting these data. Nevertheless, a sea of data and the requirements of timely diagnosis improve the complexity and difficulty of real-time fault diagnosis. Due to the introduction of feedback in the closed-loop system, when a fault occurs in one part of the system, it may cause the fault behavior to spread within the control system and make other parts abnormal. A double closed-loop exists in a cascade system, and the behaviors between the major and vice loops are closely related. Consequently, the phenomenon of fault propagation in the double closed-loop system makes fault diagnosis more complex and more difficult [5]. All parts of the cascade control system may fail in engineering applications, and the probability of sensor failure is the highest.

Fault detection and diagnosis are essential measures to improve system reliability and availability [6,7]. Numerous methods have been proposed since the development of fault detection and diagnosis technology, including roughly three categories [8,9]. First, the analytical model-based approach, for example, parameter estimation and equivalent space method [10,11], which is based on the system operation mechanism. For a large-scale system, it is arduous to establish an accurate mathematical model. Knowledge-based method as the second one, such as fault tree [12] and Expert System (ES) [13,14]. The limitation of the ES lies in relying on the domain knowledge acquisition of experts. Last but not least, the data-driven method [15,16]. The way based on data-driven is to collect, analyze and diagnose the data generated during the operation of the equipment without knowing the accurate system model. Data-driven strategies include information processing methods, statistical analysis methods, machine learning methods, et cetera. Since it can diagnose without a precise system model description, and the historical data [17,18] can be obtained entirely and sufficiently, both academia and industry attach positive importance to this method [19,20]. For instance, Rashidi et al. [21] proposed a multivariable process fault diagnosis method based on data-driven, used the normalized transfer entropy (NTE) between the measured process variables and residual signal variation to estimate the strength of causality, which reduced the amount of calculation required for analysis. Renga et al. [22] put forward a transparent, exploratory, and detailed data mining workflow based on data characterization, time window, association rule mining, and association classification. For the PEMFC system, a deep belief network (DBN) was adopted by Zhang et al. [23] to the fault diagnosis. They used the simulated annealing genetic algorithm fuzzy c-means clustering (SAGAFCM) method to eliminate redundant and invalid data. Hu et al. [24] gave a data-driven rotating machinery fault diagnosis method based on compressed sensing (CS) and an improved multi-scale network (IMSN), which can effectively identify faults under different working conditions.

In contrast with the open-loop system, the feedback effect of the closed-loop system will cause the fault to propagate within it, make other parts of the data abnormal, and reduce the performance of the system [25]. For sensors in closed-loop systems, fault detection methods of Kalman filter, parameter estimation, and maximum likelihood estimation were used by Doraiswami et al. [26]. However, they suffered from complicated calculations, time-consuming, and initialization problems. Shi [27] took the traction motor of the closed-loop system as the research object and adopted the diagnosis method based on the analytical model. However, the actual non-linear factors were not considered in the modeling process. More realistic system properties are inseparable from more precise mathematical models. Sheriff [28] adopted the fault detection methods of kernel PCA (KPCA) and kernel PLS (KPLS) to improve the accuracy, but there was still exists the false alarm and missed detection rate of more than 3%. This study proposes a real-time single fault diagnosis of sensors in a cascade system based on data-driven by studying the off-line historical data of the double tank. This method solves the shortcomings of the above techniques and has the following advantages. It calculates the collected data directly with less calculation amount, has a low missed detection rate, and well real-time performance; it does not rely on the system operating mechanism and avoids the problem of error in the modeling process.

The novelty of this research: In principle, the first is to study fault diagnosis methods according to system classification to facilitate the realization of system configuration. Here is the cascade system; Secondly, it combines real-time data, which reflects the dynamic characteristics of the system; Last is to integrate analytical geometric modeling methods. In terms of functionality, first, establishes the static models of sensor fault detection, fault location, fault estimation and fault separation in cascade system; The second is proposes a method of calibrating static models by using on-line data to obtain real-time fault diagnosis models; Then, gives the workflow and anti-interference measures of the real-time diagnosis system. This method can be applied to the single sensor fault diagnosis of the general cascade system. The effectiveness and accuracy of fault diagnosis were verified by experiments.

## 2. Structure and Characteristics of the Cascade System

**Figure 1:**
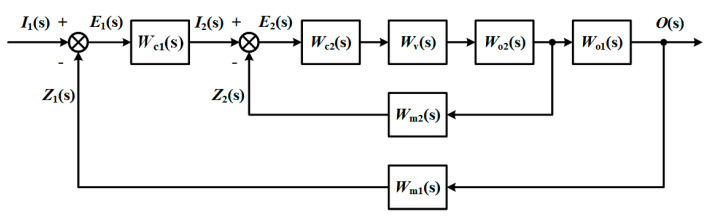
Block diagram of cascade control system.

In contrast with the single loop control system, the cascade control system has one more vice loop to form a double closed-loop structure [29] (Figure 1) which improves the dynamic characteristics of the controlled process and the ability to overcome the disturbance [30].

**Figure 2:**
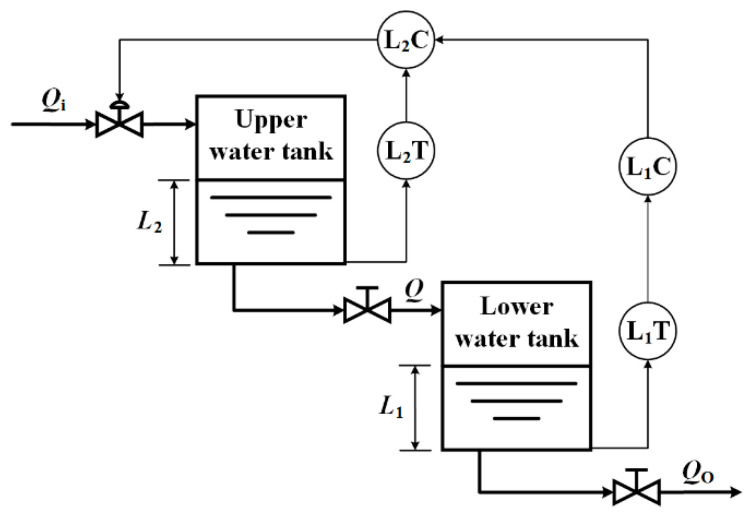
Double-capacity Water Tank Liquid Level Cascade Control System.

This study takes the Double-capacity Water Tank Level Cascade Control System (DWTLCCS) as the research object (Figure 2).

## 3. Fault Detection and Diagnosis Method

The cascade system is a double-loop system with a major loop and a vice loop. Each circle has a sensor to measure the data of the corresponding object.

Sensor faults contain many types according to different classification standards. Based on the external characteristics of the defect, the sensor faults are divided into additive fault and multiplicative fault. The symptom of additive failure is that the measured value is different from the actual value by a constant, while the sign of multiplicative failure is a constant multiple.

Sensor fault diagnosis is based on big data reflecting the dynamic characteristics of the system. Analyze the characteristic relationship of data after the system reaches control stability requirement. In the fault detection part, the data is used for fault detection in the form of windows. The following fault location, estimation, and separation diagnosis steps are performed based on the fault signs found by the fault detection.

### 3.1. Fault Detection Method

#### 3.1.1. Fault Detection Method

The fault detection algorithm is performed after the system reaches the control stability requirements. When the system is running stably, the liquid level of the water tanks will fluctuate slightly. The liquid level fluctuation refers to the slight difference between the liquid level data of two adjacent sampling points collected by sensors. It has a specific range so that the fault can be detected according to the degree of the liquid level fluctuation. Calculate the liquid level fluctuation value (LLFV) of the experimental data obtained under fault-free conditions:

(1) $$\left\{ \begin{array}{l} M_{fl}=\left|M_{c}\left(t\right)-M_{c}\left(t-T\right)\right|\\ V_{fl}=\left|V_{c}\left(t\right)-V_{c}\left(t-T\right)\right| \end{array}\right.$$

where *M_fl_* and *V_fl_* denote the LLFVs of the lower water tank (LWT) and upper water tank (UWT), respectively, *M_c_* and *V_c_* are the measured values of these two water tanks, *t* is a sampling time, and *T* is the sampling step.

Suppose that one group of experimental time is *t_a_* and liquid level adjustment time is *t_s_*. Calculate the LLFV after it has stabilized for a while *t_1_*, and the LLFV number of two water tanks is *Num*:

(2) $$Num=\frac{\left(t_{a}-t_{s}-t_{l}\right)}{T}+1$$

Carry out multi-group fault-free experiments to reduce the error and improve fault detection accuracy. Obtain the maximum LLFV of two water tanks in each group of experiments:

(3) $$\left\{ \begin{array}{l} M_{fl\max k}=\left[M_{fl\max1},M_{fl\max1},\cdots ,M_{fl\max r}\right]\\ V_{fl\max k}=\left[V_{fl\max1},V_{fl\max1},\cdots ,V_{fl\max r}\right] \end{array}\right.$$

where *r* represents the number of fault-free experimental groups. *M_fl_*_max_ and *V_fl_*_max_ stand the maximum values of the liquid level data of the LWT and UWT of each group of experiments calculated by Equation (1), respectively.

Take the LWT as the example to calculate the mean value and sample variance *σ_M_*:

(4) $${\bar{M}}_{fl\max k}=\frac{1}{r}\sum_{k=1}^{r}M_{fl\max k}$$

(5) $$\sigma_{M}=\sqrt{\frac{1}{r-1}\sum_{k=1}^{r}{\left(M_{fl\max k}-{\bar{M}}_{fl\max k}\right)}^{2}}$$

Take 3*σ* as the trade-off of abnormal data, obtain the fluctuation threshold of the LWT liquid level for primary loop sensor fault detection can as follows:

(6) $$M_{th}={\bar{M}}_{fl\max k}+3\sigma_{M}$$

Similarly, the liquid level fluctuation threshold of the UWT is:

(7) $$V_{th}={\bar{V}}_{fl\max k}+3\sigma_{V}$$

Then the static models of fault detection of the major and vice sensors are, respectively:

(8) $$M_{fl}\left(t\right)>M_{th}$$

(9) $$V_{fl}\left(t\right)>V_{th}$$

where *M_fl_* (*t*) and V*_fl_* (*t*) are the LLFVs of the LWT and UWT at a sampling time *t*, respectively.

The proposed fault detection method averaging the maximum value of multiple windows and sets of data has a certain inhibitory effect on normal sampling fluctuations in engineering.

#### 3.1.2. Interference Signal Suppression

Consider the fault detection method in this study is to process the instantaneous value of system running data, so suppressing the interference signal to avoid misjudgment is essential.

For random data burr interference, restrain it during the fault detection process to realize the operation of suppressing interference while detecting. The LLFV in both cases of fault and interference signal will exceed the corresponding threshold. The difference is as follows: in the former, only a single LLFV exceeds the corresponding threshold, namely, its adjacent LLFVs are all below the threshold; in the latter, at least two adjacent LLFVs exceed the corresponding threshold. We can distinguish between a system failure and an interference signal according to the difference between the two. For other disturbances such as system and environment, the fault detection method uses the measure of taking the average value, which itself has a certain function of restraining interference. For disturbance has a linear relationship with the fault, the adjustment period after the defect is used to correct the level data to achieve suppression. The disturbance and the fault are distinguished by the adjustment period.

### 3.2. Fault Location Method

#### 3.2.1. Fault Location of Major Loop Sensor

The liquid level of LWT is the controlled variable, the loop it is in is the main loop, and its sensor is the major loop sensor. When the sensor in the major loop fails, the measured value of the sensor will change abruptly, and the LLFV in the lower water tank will be greater than the threshold. Through the feedback of the cascade system, the vice loop will tailor according to the measured data for the major loop sensor. In some cases, the liquid level of the UWT over-adjustment may also induce its LLFV to exceed the threshold.

Through the above analysis, we can infer that the judgment conditions of sensor failure in the major loop are as follows:

(10) $$M_{fl}\left(t_{MF}\right)>M_{th}$$

(11) $$\left\{ \begin{array}{l} M_{fl}\left(t_{MF}\right)>M_{th}\\ V_{fl}\left(t_{VF}\right)>V_{th} \end{array}\right.,\;and\;t_{MF}<t_{VF}$$

Meeting either of the above conditions represents the system fault located in the major loop sensor. Where *t_MF_* and *t_VF_* are the sampling times when the fluctuation value of LWT and UWT exceeds the corresponding threshold, respectively.

#### 3.2.2. Fault Location of Vice Loop Sensor

When the vice loop sensor fails, the measured value of the sensor will change abruptly, and the current fluctuation value of the UWT will exceed the threshold. The fault of the vice sensor is almost no influence on it if the liquid level in the LWT has reached a stable state at this time. Hence, the LLFV in the LWT will not change significantly and will remain below the threshold.

Thus, the judgment condition for the failure of the vice loop sensor is as follows:

(12) $$V_{fl}\left(t_{VF}\right)>V_{th}$$

If the above condition is satisfied, the fault occurs in the vice loop sensor.

### 3.3. Fault Estimation Method

Fault estimation consists of the estimation for fault occurrence time and intensity.

#### 3.3.1. Fault Time Estimation

Combine the fault detection and fault location algorithm to determine the time of the failure.

When the major loop sensor fails, the LLFV in the LWT at the failure sampling moment can be known by the fault detection algorithm, and then the fault occurrence time is known as *t_MF_* by the fault location algorithm Formulas (10) and (11). Similarly, the fault time is *t_VF_* described in Formula (12) when the vice loop sensor fails.

#### 3.3.2. Fault Intensity Estimation

The fault intensity is the comparison between the liquid level sampling value at the time of failure and the one before the failure.

When the major loop sensor generates an additive fault, the fault intensity *M_Fp_* is the result of the sampling value at the fault time minus the value at the previous time:

(13) $$M_{Fp}=M_{c}\left(t_{MF}\right)-M_{c}\left(t_{MF}-T\right)$$

When the major loop sensor has a multiplicative fault, the fault intensity *M_Fm_* is the result of dividing the sampling value at the fault time by the value at the previous time:

(14) $$M_{Fm}=\frac{M_{c}\left(t_{MF}\right)}{M_{c}\left(t_{MF}-T\right)}$$

Similarly, when the vice loop sensor has an additive fault, the fault intensity *V_Fp_* is:

(15) $$V_{Fp}=V_{c}\left(t_{VF}\right)-V_{c}\left(t_{VF}-T\right)$$

When the vice loop sensor occurs a multiplicative fault, the fault intensity *V_Fm_* is:

(16) $$V_{Fm}=\frac{V_{c}\left(t_{VF}\right)}{V_{c}\left(t_{VF}-T\right)}$$

The above equations can estimate the additive and multiplicative fault intensities of the major and vice sensors.

### 3.4. Fault Separation Method

Fault separation is to analyze the properties of the faults detected by the above methods and judge whether the faults belong to additive fault or multiplicative fault.

#### 3.4.1. Fault Separation of Major Loop Sensor

After failure, the measured level of LWT will mutate and then change in the opposite direction to the fault until it returns to a stable state again. In this process, the controller will adjust the liquid level for UWT according to the deviation degree of the liquid level for LWT. There exists a specific relationship between the liquid level changes of two water tanks. Consider the different states on the dynamic relationship due to different types and intensities of failures, regard the changing data as the preliminary characteristic data set. Then the measured data of the lower and upper water tank as independent and dependent variables, respectively, the first non-linear regression analysis of the characteristic data set is carried out by using Equation (17) to obtain a smooth characteristic data set:

(17) $$Y_{1}=\alpha_{1}M^{2}+\alpha_{2}M+\alpha_{3}+\varepsilon$$

Select multiple groups of data with different failure types and intensities for regression analysis according to Equation (17). These curves are equivalent to parabolas with different coefficients and represent the liquid level change trace after different types and intensities faults. Therefore, one of the three elements of the parabola, namely the axis of symmetry, can be used as the fault eigenvalue. According to the symmetry axis formula:

(18) $$S=-\frac{\alpha_{2}}{2\alpha_{1}}$$

We can acquire the eigenvalue set *S_P_* of additive fault is:

(19) $$S_{P}={\left[p_{11},p_{12},p_{13},\cdots \right]}^{T}$$

where, *p*_11_, *p*_12_, *p*_13_, … represent the characteristic values of the symmetry axis under different strength additive faults. The eigenvalue set *S_M_* of multiplicative fault is:

(20) $$S_{M}={\left[m_{11},m_{12},m_{13},\cdots \right]}^{T}$$

where, *m*_11_, *m*_12_, *m*_13_, …represent the characteristic values of the symmetry axis under different strength multiplicative faults. After selecting the fault eigenvalue *S*, the second non-linear regression analysis uses the least square principle. The independent and dependent variables are fault intensity and eigenvalue *S*, respectively. Thus, we can acquire the fault separation model of the major loop sensor.

The additive fault model is:

(21) $$S_{p}=f\left(M_{Fp}\right)$$

The multiplicative fault model is:

(22) $$S_{m}=g\left(M_{Fm}\right)$$

Finally, compare the real-time fault eigenvalue *S* with the *Sp* and *Sm* obtained by the above fault separation models:

(23) $$\left\{ \begin{array}{l} e_{1}=\left|S-S_{p}\right|\\ e_{2}=\left|S-S_{m}\right| \end{array}\right.$$

If *e*_1_ < *e*_2_, it is judged that the major loop sensor generates an additive fault; otherwise, multiplicative fault.

#### 3.4.2. Fault Separation of Vice Loop Sensor

After the vice loop sensor fails, the direction of liquid level change is completely different from the failure. At this time, the liquid level of the LWT has reached a stable state, so the change has little impact on it. Therefore, the characteristic data set only includes the data of UWT with apparent changes. Pay significant concern on the different initial change rates feature data for the UWT among various faults. Smooth the characteristic data set according to:

(24) $$Y_{2}=\beta_{1}V+\beta_{2}+\varepsilon$$

Select multiple groups of data with different failure types and intensities for regression analysis according to Equation (24) to obtain a series of primary linear functions with different coefficients. The coefficients of each primary term are regarded as fault eigenvalues and bring the following results:

The eigenvalue set *SL_P_* of additive fault is:

(25) $$SL_{P}={\left[p_{21},p_{22},p_{23},\cdots \right]}^{T}$$

where, *p*_21_, *p*_22_, *p*_23_, … represent the characteristic values of the symmetry axis under different strength additive faults. The eigenvalue set *SL_M_* of multiplicative fault is:

(26) $$SL_{M}={\left[m_{21},m_{22},m_{23},\cdots \right]}^{T}$$

where, *m*_21_, *m*_22_, *m*_23_, … represent the characteristic values of the symmetry axis under different strength multiplicative faults. The additive fault model is:

(27) $$SL_{p}=q\left(V_{Fp}\right)$$

The multiplicative fault model is:

(28) $$SL_{m}=y\left(V_{Fm}\right)$$

Finally, compare the real-time coefficient of the first-order term with the *SL_p_* and *SL_m_* obtained by the above fault separation models:

(29) $$\left\{ \begin{array}{l} e_{3}=\left|\beta_{1}-SL_{p}\right|\\ e_{4}=\left|\beta_{1}-SL_{m}\right| \end{array}\right.$$

The judgment method is the same concept as that of the major loop sensor. That is, the valid result is the one with a smaller value.

### 3.5. On-Line Fault Diagnosis Algorithm Flow

Static models of fault diagnosis are given above. In practical applications, calibrate can convert the static model into a dynamic model. Calibration is based on the static model, and the steps are as follows: (a) combine the eigenvalues of historical data for different faults; (b) obtain various parameters through the non-linear least square principle; (c) acquire the data change law caused by faults suitable for the states of various components of different engineering cascade systems; (d) complete the calibration.

**Figure 3:**
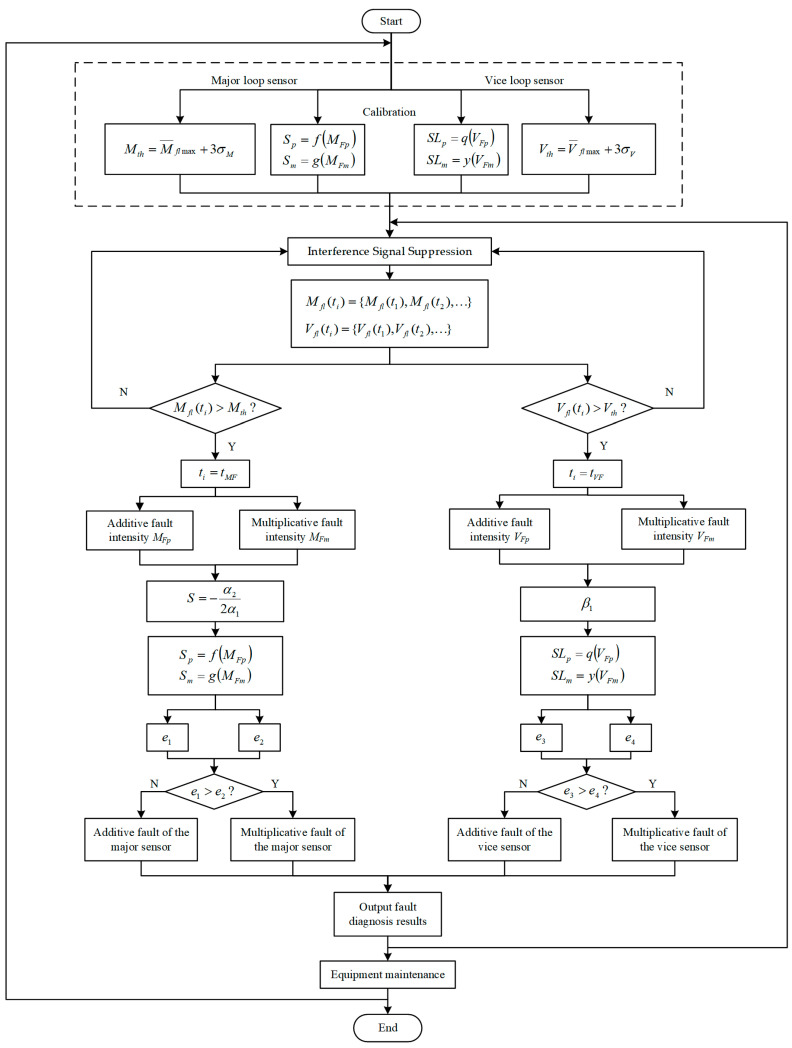
Flowchart of on-line fault diagnosis.

We acquire the on-line fault diagnosis of practical engineering by algorithms and calibration method above. The diagnosis steps in the sequence are fault detection and interference signal elimination, fault location, fault estimation, and separation. Figure 3 represents the on-line calibration and diagnosis process.

## 4. Verification of Fault Detection and Diagnosis Method

### 4.1. Experiment Platform

The research is based on the “complex system fault diagnosis and fault-tolerant control innovation platform,” which is composed of device hardware and PC software. Hardware composition: a typical process control object—four-capacity water tank; ultrasonic sensor with a range of 0–1 m, a blind area less than 0.06 m, and a precision and minimum display resolution of 1 mm; DC water pump; Burkert proportional solenoid valve, composed of 6223 valve body and 8065 controller; PLC part uses Siemens S7-300 module introduced at the end of the 20th century. Software part: WinCC upper computer configuration software; Matlab (2015b)/Simulink; STEP7 for configuration programming of PLC independent hardware unit control mechanism. Communication part: Simatic Net (Manufacturer: Simens AG, Berlin, Germany), Siemens industrial communication solution for OPC (OLE for Process Control) to realize data integration in the control system, realize data communication.

**Figure 4:**
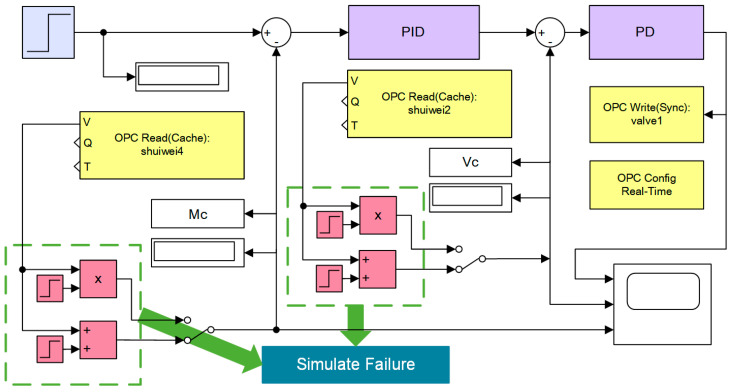
Experimental model in Simulink.

The experimental model is created with Matlab/Simulink library browser, as shown in Figure 4. The input signal is a step signal. The primary controlled object control requires no residual error, so the PID control law. The secondary allows the residual error and speeds up the adjustment time, so the PD control law. Rely on the system characteristics and empirical method, the main controller parameters are set as P = 11.9, I = 0.07, D = 16.8; the secondary controller P = 1, D = 5.8. Each closed loop has an analog additive and multiplicative fault occurrence module, which can customize the occurrence time and intensity.

### 4.2. Data Acquisition

Divide the experimental data into five states: fault-free state, additive fault states on major and vice loop sensors, multiplicative fault states on two sensors.

During the experiment, the expected liquid level of the lower water tank was 10 cm, the experimental time was 1200 s, and the sampling step *T* was 0.5 s.

#### 4.2.1. Fault-Free Data Acquisition

**Figure 5:**
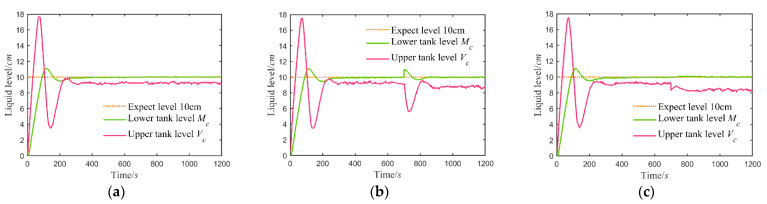
The liquid level curves: (**a**) in the fault-free state; (**b**) in the additive fault (−1.0 cm) state for the major loop sensor; (**c**) in the multiplicative fault (0.9) state for the vice loop sensor.

We collected multiple groups of fault-free data to reduce error. Set the expected liquid level of each group of the fault-free experiment at 10 cm. The experimental running time was 1200 s, and the sampling step was 0.5 s. Figure 5a shows the system liquid level response curve for one group of fault-free experiments in the form of time series, including the expected liquid level of the main controlled object and the original data of two water tanks directly obtained by the sensors.

#### 4.2.2. Fault Data Acquisition

Maintained the control parameters in the fault-free state, then added additive and multiplicative faults of different intensities to two sensors at 700 s after the system ran to a stable condition. Set the range of additive fault intensity between ±2.0 cm and multiplicative in 0.8~1.2. Take the additive fault (−1.0 cm) of the sensor in the major loop and the multiplicative fault (0.90) in the vice loop as examples, the system liquid level response curves as shown in Figure 5b,c by the form of time series, respectively. The expected liquid level of the main controlled object and the original liquid level data of LWT and UWT directly obtained by the sensors in the case of failure are drawn in the figure.

The additive fault in Figure 5b occurs in the primary loop sensor, which measures the liquid level of LWT. The multiplicative defect in Figure 5c arises in the secondary loop sensor to obtain the data of UWT. LWT is the main control object of the cascade system in this paper. When it is not at the set value, such as the additive fault of the sensor measuring the lower water tank here, the controller will adjust the actuator to increase or reduce the water inflow. This step acts directly on UWT, which will change the liquid level of itself. If LWT has reached the expected value and stabilized, a slight change in the liquid level of the upper at this time, such as Figure 5c, will have little effect on LWT. The reason is that the volume of UWT affects the buffering impact of its liquid level and the timely adjustment of the controller.

### 4.3. Verification of Fault Detection Method

Taking 10 groups data of fault-free, we acquired the average and sample variance of the maximum LLFVs of the LWT and UWT after the system reaches the control stability requirements, respectively, according to Equations (4) and (5):

$$\begin{array}{l} {\bar{M}}_{fl\max}=0.1085\;\mathrm{cm},\;\sigma_{M}=0.0147\;\mathrm{cm}\\ \;{\bar{V}}_{fl\max}=0.1216\;\mathrm{cm},\;\sigma_{V}=0.0115\;\mathrm{cm} \end{array}$$

Then, the liquid level fluctuation thresholds of the two water tanks were calculated by the Equations (6) and (7):

$$\begin{array}{l} M_{th}={\bar{M}}_{fl\max}+3\sigma_{M}=0.1526\;\mathrm{cm}\\ \;V_{th}={\bar{V}}_{fl\max}+3\sigma_{V}=0.1563\;\mathrm{cm} \end{array}$$

After the system runs stably, run the fault detection algorithm in real-time based on the collected data. The detection results of faults within ±3% through the above thresholds are listed in Table 1 and Table 2 (0: fault-free; 1: out of order). The deviation and gain in the tables represent the faults intensity within ±3%, respectively.

Tables indicate that failure intensities beyond 1.6% on the major sensor and beyond 2% on the vice sensor did not appear missed detection.

### 4.4. Verification of Fault Location Method

#### 4.4.1. Fault Location of Major Loop Sensor

**Figure 6:**
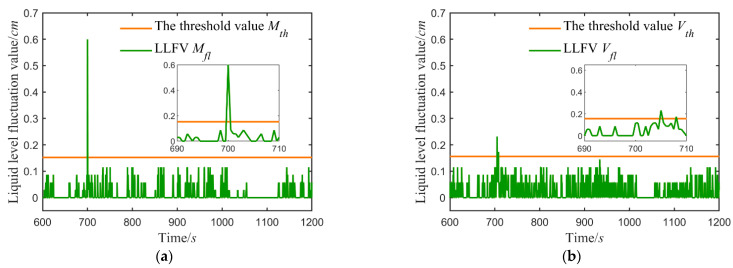
Additive fault on the major loop sensor is +0.6 cm: (**a**) the LLFV of the lower water tank; (**b**) the LLFV of the upper water tank.

After the system is stable, the fault before, with the help of Equation (1), in the form of moving small Windows, calculated each sample point is corresponding to the liquid level fluctuation. The LLFVs of the upper and lower water tanks were calculated. Take the case of the additive fault +0.6 cm, as shown in Figure 6.

Figure 6 shows the LLFVs of the two water tanks both exceed their threshold, but LWT exceeds the threshold value earlier than UWT, which satisfies In Equation (11). Consequently, the sensor in the major loop has failed.

The location results of faults within ±6% on the major loop sensor are listed in Table 3 (1: major loop sensor; 2: vice loop sensor), showing the location accuracy is 100%.

#### 4.4.2. Fault Location of Vice Loop Sensor

**Figure 7:**
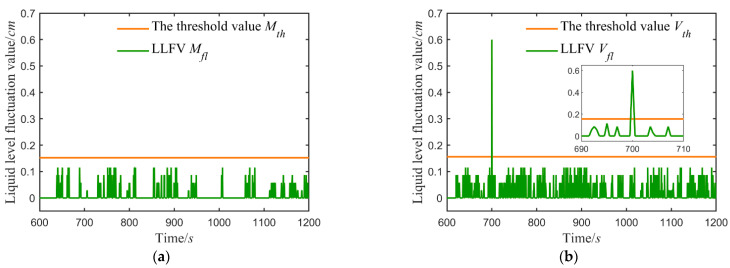
Additive fault on the vice loop sensor is +0.6 cm: (**a**) the LLFV of the lower water tank; (**b**) the LLFV of the upper water tank.

Take the additive fault +0.6 cm as an example, calculated the liquid level fluctuation values, as shown in Figure 7.

Figure 7 indicates the LLFV of LWT remains below the threshold, and only that of UWT exceeds the threshold, which matches the In Equation (12). Thus, the failed sensor in the vice loop.

Table 4 represents the location results of faults within ±6% of the vice loop sensor (1: major loop sensor; 2: vice loop sensor).

The outcome indicates that the fault location method can effectively judge the location of additive fault with 100% accuracy. It can also locate multiplicative faults beyond 2% accurately.

### 4.5. Verification of Fault Estimation Method

#### 4.5.1. Fault Estimation of Major Loop Sensor

Take the additive fault +0.6 cm as an example, and Figure 6 represents the fault that occurs at 700 s:

$$\left\{ \begin{array}{l} M_{fl}\left(t_{MF}\right)>M_{th}\\ V_{fl}\left(t_{VF}\right)>V_{th} \end{array}\right.,\;\left\{ \begin{array}{l} t_{MF}=700\;s,\;t_{VF}=705\;s\\ t_{MF}<t_{VF} \end{array}\right.$$

Take two kinds of fault data within the range of ±6% to verify by the fault estimation algorithm, and the verification results are shown in Table 5 and Table 6.

#### 4.5.2. Fault Estimation of Vice Loop Sensor

Take the additive fault +0.6 cm for instance, and Figure 6 shows the fault that occurs at 700 s:

$$V_{fl}\left(t_{VF}\right)>V_{th},and\;\;t_{VF}=700\;s$$

Take two kinds of fault data within the range of ±6% to verify by the fault estimation algorithm, and the verification results are shown in Table 7 and Table 8.

By calculation, the relative errors of additive and multiplicative faults for the major loop sensor are 0% and 0.29%, respectively. Both are 0% for the vice loop sensor.

### 4.6. Verification of Fault Separation Method

#### 4.6.1. Fault Separation of Major Loop Sensor

**Figure 8:**
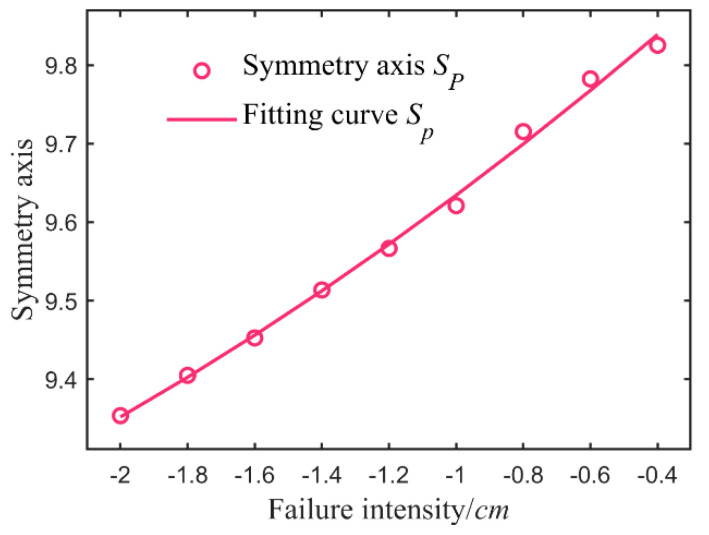
Feature fitting of major loop sensor additive faults < 0.

It is essential to acquire a batch of experimental data on fault states with different intensities to establish the fault separation static models for the major loop sensor. Based on the liquid level adjustment data of two tanks within a period of time after the fault, the eigenvalues corresponding to different fault intensities of additive and multiplicative—axis of symmetry were calculated by Equations (17) and (18). The second row of Table 9, Table 10, Table 11 and Table 12 lists the eigenvalue sets obtained—Equations (19) and (20). Table 9, for example, obtained a fitting curve (Figure 8)—Equation (21), reflecting the relationship between failure intensity and characteristic value, from the characteristic data with an outlier removed of additive fault *M_Fp_* < 0. Similarly, data in Table 10, Table 11 and Table 12 were analyzed by the second non-linear regression—the fitting functions are listed in Table 13.

Table 14 and Table 15 indicate the verification results of fault separation for the major loop sensor. The accuracy of fault separation reaches 5%.

#### 4.6.2. Fault Separation of Vice Loop Sensor

**Figure 9:**
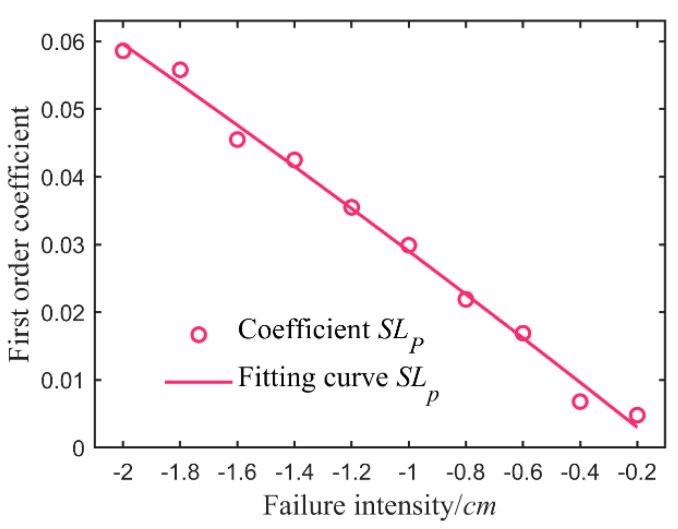
Feature fitting of vice loop sensor additive faults < 0.

A batch of experimental data with different fault strengths was taken to establish the qualitative static fault model for each type of fault. According to Equation (24), the characteristic values corresponding to different additive and multiplicative fault intensities-primary term coefficients of liquid level adjustment data of UWT after failure are calculated. The second row in Table 16, Table 17, Table 18 and Table 19 shows the obtained set of eigenvalues—Equations (25) and (26). Take Table 16 as an example, bringing the fitting curve (Figure 9)—Equation (27), reflecting the connection between failure intensity and characteristic value, from the characteristic data with the additive fault deviation *V_Fp_* < 0. Similarly, the second non-linear regression was analyzed according to eigenvalues in Table 17, Table 18 and Table 19—the fitting functions listed in Table 20.

Table 21 and Table 22 indicate the verification results of fault separation for the vice loop sensor. The accuracy of fault separation reaches 7%.

Further verified by a large amount of data: for the major loop sensor, the dead zone of fault detection is 1.6%, fault location accuracy is 100%, the relative error of fault estimation is 0.29%, 5% dead zone for fault separation; for the sensor in the vice loop, the dead zone of fault detection is 2%, 100% accuracy for fault location, no relative error in fault estimation, and the dead zone of fault separation is 7%.

## 5. Conclusions

In this study, we have taken the major and vice loop sensors of DWTLCCS as the analysis objects. The static models of system fault detection, location, estimation, and separation are established via historical data, which has characteristic information generated by the system operation. On balance, based on the data-driven method. We proposed the measurement to suppress disturbance, giving the calibration method and on-line fault diagnosis process. Experiments verified the effectiveness of the fault diagnosis method. The method is universal for single fault diagnosis of sensors in the cascade control system.

The traditional detection threshold is commonly determined based on the sum of residuals. A clear expectation value is required for this method. Since the vice loop of the cascade system is not the primary control object, and its controller plays the role of serving control and does not need to use integral control, which will generate the steady-state error. In such a case, the liquid level of the secondary has no definite expected value. Suppose calculated threshold value in the form of residual error sum will cause missed or false detection. The detection method we proposed reduces the missed detection rate and the amount of calculation, improving the detection accuracy simultaneously.

The detection threshold is determined according to the method put forward in this paper, and the actual system calibrates the on-line diagnosis model. To some extent, it has a certain inhibitory effect on noise, and higher detection accuracy is obtained. How to further suppress the disturbance effect is the future research focus.

Based on system dynamic characteristics and data mining methods, this study adopts the analytical geometry approach to modeling, fully utilizing data information. A precise mathematical model of the system is needless and analyzes the data generated by the system operation in real-time directly, avoiding the issue of modeling errors that depend on the mathematical model. Diagnosis is carried out in real-time, without an obvious delay problem.

During operation, considering the wear and tear of system components, regular calibration can be carried out to ensure the accuracy of diagnosis. In future work, we plan to investigate further how to enhance diagnosis precision.

## Figures and Tables

**Table 1:** Detection results of faults within ±6% of major sensor.

**Additive Fault**	**Deviation/cm**	−0.3	−0.2	−0.16	−0.1	+0.1	+0.16	+0.2	+0.3
	**Result**	1	1	1	0	0	1	1	1
**Multiplicative Fault**	**Gain**	0.97	0.98	0.984	0.99	1.01	1.016	1.02	1.03
	**Result**	1	1	1	0	0	1	1	1

**Table 2:** Detection results of faults within ±6% of vice sensor.

**Additive Fault**	**Deviation/cm**	−0.3	−0.2	−0.16	−0.1	+0.1	+0.16	+0.2	+0.3
	**Result**	1	1	1	0	0	1	1	1
**Multiplicative Fault**	**Gain**	0.97	0.98	0.984	0.99	1.01	1.016	1.02	1.03
	**Result**	1	1	0	0	0	0	1	1

**Table 3:** Location results of faults within ±6% of major sensor.

**Additive Fault**	**Deviation/cm**	−0.5	−0.4	−0.3	−0.2	−0.16	+0.16	+0.2	+0.3	0.4	0.5
	**Result**	1	1	1	1	1	1	1	1	1	1
**Multiplicative Fault**	**Gain**	0.95	0.96	0.97	0.98	0.984	1.016	1.02	1.03	1.04	1.05
	**Result**	1	1	1	1	1	1	1	1	1	1

**Table 4:** Location results of faults within ±6% of vice sensor.

**Additive Fault**	**Deviation/cm**	−0.5	−0.4	−0.3	−0.2	−0.16	+0.16	+0.2	+0.3	0.4	0.5
	**Result**	2	2	2	2	2	2	2	2	2	2
**Multiplicative Fault**	**Gain**	0.95	0.96	0.97	0.98	0.984	1.016	1.02	1.03	1.04	1.05
	**Result**	2	2	2	2	/	/	2	2	2	2

**Table 5:** Estimated results of additive faults within ±6% of major sensor.

Deviation/cm	Fault Estimation/cm	Error|*Ea*|/cm
−0.5	−0.5	0
−0.4	−0.4	0
−0.3	−0.3	0
−0.2	−0.2	0
−0.16	−0.16	0
0.16	0.16	0
+0.2	+0.2	0
+0.3	+0.3	0
+0.4	+0.4	0
+0.5	+0.5	0

**Table 6:** Estimated results of multiplicative faults within ±6% of major sensor.

Gain	Fault Estimation	Error|*Em*|
0.95	0.95	0
0.96	0.96	0
0.97	0.9672	0.0028
0.98	0.98	0
0.984	0.9812	0.0028
1.016	1.016	0
1.02	1.02	0
1.03	1.036	0.006
1.04	1.04	0
1.05	1.05	0

**Table 7:** Estimated results of additive faults within ±6% of the vice sensor.

Deviation/cm	Fault Estimation/cm	Error|*Ea*|/cm
−0.5	−0.5	0
−0.4	−0.4	0
−0.3	−0.3	0
−0.2	−0.2	0
−0.16	−0.16	0
0.16	0.16	0
+0.2	+0.2	0
+0.3	+0.3	0
+0.4	+0.4	0
+0.5	+0.5	0

**Table 8:** Estimated results of multiplicative faults within ±6% of the vice sensor.

Gain	Fault Estimation	Error|*Em*|
0.95	0.95	0
0.96	0.96	0
0.97	0.97	0
0.98	0.98	0
0.984	/	/
1.016	/	/
1.02	1.02	0
1.03	1.03	0
1.04	1.04	0
1.05	1.05	0

**Table 9:** The symmetry axis of the preliminary fitting function for additive fault deviation < 0.

Deviation *M_Fp_* < 0	−0.2	−0.4	−0.6	−0.8	−1.0	−1.2	−1.4	−1.6	−1.8	−2.0
Symmetry axis *S_P_*	Abnormal	9.8253	9.7824	9.7153	9.621	9.5665	9.5136	9.4526	9.4049	9.3536
RMSE	/	0.1030	0.1338	0.1394	0.1476	0.1298	0.1914	0.1917	0.2237	0.2098

**Table 10:** The symmetry axis of the preliminary fitting function for additive fault deviation > 0.

Deviation *M_Fp_* > 0	0.2	0.4	0.6	0.8	1.0	1.2	1.4	1.6	1.8	2.0
Symmetry axis *S_P_*	10.1021	10.2322	10.2867	10.2867	10.4100	9.5665	9.5136	10.6346	10.7780	10.8680
RMSE	0.1482	0.1850	0.2048	0.1639	0.1463	0.1641	0.1777	0.1203	0.1670	0.1678

**Table 11:** The symmetry axis of preliminary fitting function for multiplicative fault gain < 1.

Gain *M_Fm_* < 1	0.98	0.96	0.94	0.92	0.90	0.88	0.86	0.84	0.82	0.80
Symmetry axis *S_M_*	9.8963	9.8578	9.7687	9.7180	9.6428	9.6057	9.5437	9.4762	9.2899	9.2289
RMSE	0.1083	0.1039	0.1329	0.1557	0.1511	0.1846	0.2252	0.2209	0.2243	0.2526

**Table 12:** The symmetry axis of preliminary fitting function for multiplicative fault gain > 1.

Gain *M_Fm_* > 1	1.02	1.04	1.06	1.08	1.10	1.12	1.14	1.16	1.18	1.20
Symmetry axis *S_M_*	10.0984	10.2303	10.2423	10.3671	10.4737	10.5825	10.6813	10.7172	10.8310	10.8631
RMSE	0.1492	0.1673	0.1448	0.1572	0.1814	0.1736	0.1874	0.1535	0.1275	0.1500

**Table 13:** Results of the second non-linear regression analysis.

Fault Type	Fitting Function of the Second Nonlinear Regression	*r* ^2^	RMSE
*M_Fp_* < 0	$S_{p}=0.0368M_{Fp}{}^{2}+0.3926M_{Fp}+9.9900$	0.9961	0.0121
*M_Fp_* > 0	$S_{p}=0.0542M_{Fp}{}^{2}+0.2475M_{Fp}+10.0867$	0.9854	0.0307
*M_Fm_* < 1	$S_{m}=-8.2140M_{Fm}{}^{2}+18.2708M_{Fm}-0.1268$	0.9939	0.0198
*M_Fm_* > 1	$S_{m}=-6.2519M_{Fm}{}^{2}+17.4998M_{Fm}-1.2400$	0.9892	0.0261

**Table 14:** Separation results of additive faults of the major sensor.

Deviation *M_Fp_*/cm	*e*1	*e*2	Result
−0.9	0.0076	0.0230	Additive
−0.7	0.0026	0.0244	Additive
−0.5	0.0023	0.0119	Additive
−0.3	0.0282	0.0199	Multiplicative
−0.16	0.02945	0.3240	Additive
0.16	0.0828	0.0379	Multiplicative
0.3	0.0165	0.0364	Additive
0.5	0.00084	0.0140	Additive
0.7	0.00046	0.0353	Additive
0.9	0.0020	0.0537	Additive

**Table 15:** Separation results of multiplicative faults of the major sensor.

Gain *M_Fm_*	*e*1	*e*2	Result
0.91	0.0299	0.00058	Multiplicative
0.93	0.0150	0.0120	Multiplicative
0.95	0.0117	0.0023	Multiplicative
0.97	0.0017	0.0026	Additive
0.984	0.0087	0.0332	Additive
1.016	0.3279	0.2829	Multiplicative
1.03	0.0328	0.0243	Multiplicative
1.05	0.0092	0.0022	Multiplicative
1.07	0.0242	0.0134	Multiplicative
1.09	0.0302	0.0260	Multiplicative

**Table 16:** First order coefficient of additive fault deviation < 0.

Deviation *V_Fp_* < 0	−0.2	−0.4	−0.6	−0.8	−1.0	−1.2	−1.4	−1.6	−1.8	−2.0
Coefficient *SL_P_*	0.0048	0.0068	0.0169	0.0219	0.0299	0.0355	0.0425	0.0455	0.0558	0.0586
RMSE	0.0434	0.0171	0.0324	0.0377	0.0357	0.0291	0.0557	0.0459	0.0483	0.0550

**Table 17:** First order coefficient of additive fault deviation > 0.

Deviation *V_Fp_* > 0	0.2	0.4	0.6	0.8	1.0	1.2	1.4	1.6	1.8	2.0
Coefficient *SL_P_*	Abnormal	−0.0105	−0.0113	−0.0142	−0.0190	−0.0283	−0.0341	−0.0441	−0.0615	−0.0702
RMSE	/	0.0152	0.0159	0.0251	0.0206	0.0610	0.0624	0.0310	0.0222	0.0431

**Table 18:** First order coefficient of multiplicative fault gain < 1.

Gain *V_Fm_* < 1	0.98	0.96	0.94	0.92	0.90	0.88	0.86	0.84	0.82	0.80
Coefficient *SL_M_*	Abnormal	0.0058	0.0125	0.0145	0.0225	0.0294	0.0368	0.0386	0.0430	0.0474
RMSE	/	0.0185	0.0146	0.0530	0.0341	0.0236	0.0291	0.0302	0.0279	0.0313

**Table 19:** First order coefficient of multiplicative fault gain > 1.

Gain *V_Fm_* > 1	1.02	1.04	1.06	1.08	1.10	1.12	1.14	1.16	1.18	1.20
Coefficient *SL_M_*	−0.0107	−0.0077	−0.0159	−0.0186	−0.0273	−0.0357	−0.0458	−0.0567	−0.0630	−0.0753
RMSE	0.0573	0.01718	0.0326	0.0192	0.0370	0.03329	0.0554	0.03639	0.0454	0.0502

**Table 20:** Results of the second non-linear regression analysis.

Fault Type	Fitting Function of the Second Nonlinear Regression	*r* ^2^	RMSE
*V_Fp_* < 0	$SL_{p}=\;-0.0011V_{Fp}{}^{2}-\;0.0399V_{Fp}-\;0.0037$	0.9926	0.0019
*V_Fp_* > 0	$SL_{p}=-0.0226V_{Fp}{}^{2}+0.0155V_{Fp}-0.0126$	0.9939	0.0020
*V_Fm_* < 1	$SL_{m}=-0.3864V_{Fm}{}^{2}+0.4103M_{Fm}-0.0354$	0.9884	0.0018
*V_Fm_* > 1	$SL_{m}=-1.3220V_{Fm}{}^{2}+2.5522M_{Fm}-1.2354$	0.9915	0.0025

**Table 21:** Separation results of additive faults of the vice sensor.

Deviation *V_Fp_*/cm	*e*3	*e*4	Result
−0.9	0.0005	0.0039	Additive
−0.7	0.0002	0.0029	Additive
−0.5	0.0127	0.0088	Multiplicative
−0.3	0.0090	0.0130	Additive
−0.16	0.0146	0.0105	Multiplicative
0.16	0.0119	0.0100	Multiplicative
0.3	0.0070	0.0065	Multiplicative
0.5	0.0019	0.0014	Multiplicative
0.7	0.0006	0.0059	Additive
0.9	0.0040	0.0056	Additive

**Table 22:** Separation results of multiplicative faults of the vice sensor.

Gain *V_Fm_*	*e*3	*e*4	Result
0.91	0.0033	0.0002	Multiplicative
0.93	0.0025	0.0008	Multiplicative
0.95	0.0069	0.0038	Multiplicative
0.97	0.0047	0.0087	Additive
0.984	/	/	/
1.016	/	/	/
1.03	0.0077	0.0068	Multiplicative
1.05	0.0010	0.0017	Additive
1.07	0.0105	0.0051	Multiplicative
1.09	0.0092	0.0054	Multiplicative

## Data Availability

The data presented in this study are available on request from the corresponding author. The data are not publicly available due to [privacy].
